# Sarcopenia and Vitamin D Deficiency in Patients with Crohn’s Disease: Pathological Conditions That Should Be Linked Together

**DOI:** 10.3390/nu13041378

**Published:** 2021-04-20

**Authors:** Francesco Palmese, Rossella Del Toro, Giulia Di Marzio, Pierluigi Cataleta, Maria Giulia Sama, Marco Domenicali

**Affiliations:** 1 Department of Internal Medicine, AUSL della Romagna, S. Maria delle Croci Hospital, 48121 Ravenna, Italy; palmesefrancesco@gmail.com (F.P.); rossella.deltoro@auslromagna.it (R.D.T.); pierluigi.cataleta@auslromagna.it (P.C.); mariagiulia.sama@auslromagna.it (M.G.S.); 2 Endocrinology and Diabetes Unit, Campus Bio-Medico University of Rome, 00128 Rome, Italy; 3 Department of Experimental and Clinical Medicine, University of Florence, Careggi University Hospital, 50139 Florence, Italy; giulia.dimarzio@unifi.it; 4 Department of Medical and Surgical Sciences, Alma Mater Studiorum University of Bologna, 40138 Bologna, Italy

**Keywords:** sarcopenia, vitamin D, Crohn’s disease, inflammatory bowel disease, nutritional assessment, malnutrition, skeletal muscle function

## Abstract

Sarcopenia is a prevalent condition in patients with Crohn’s disease (CD), representing an independent predictor factor for the development of major postoperative complications. Thus, a proper assessment of the muscle strength, by using different validated tools, should be deemed an important step of the clinical management of these patients. Patients with CD are frequently malnourished, presenting a high prevalence of different macro- and micro-nutrient deficiencies, including that of vitamin D. The available published studies indicate that vitamin D is involved in the regulation of proliferation, differentiation, and regeneration of muscle cells. The relationship between vitamin D deficiency and sarcopenia has been extensively studied in other populations, with interesting evidence in regards to a potential role of vitamin D supplementation as a means to prevent and treat sarcopenia. The aim of this review was to find studies that linked together these pathological conditions.

## 1. Introduction

Crohn’s disease (CD) is a chronic and progressive inflammatory bowel disease (IBD) that has a high impact on a patient’s quality of life. It is well known that all segments of the gastrointestinal tract can be affected by CD, mainly the terminal ileum and colon. Inflammation is generally segmental, asymmetrical, and transmural [1]. Although progress has been made to achieve prolonged remission, almost half of the patients over time will develop complications (i.e., strictures, fistulas, and abscesses) that require surgical treatments [2]. The pathogenesis of CD is not yet fully understood, however, it clearly involves multiple factors, i.e., genetic susceptibility, environmental factors, and intestinal microflora, resulting in dysregulation of multiple and overlapping immune pathways [1].

In the last decades, the prevalence of CD has increased continuously worldwide, especially in the developed countries, primarily due to environmental factors, such as changes in dietary patterns and alterations in body composition [1]. Among these, the loss of muscle mass resulting in a decrease of muscle strength, a condition named sarcopenia, is an increasingly prevalent condition in patients with CD and is a strong independent predictor factor for the appearance of major postoperative complications [3].

Nowadays, it is widely acknowledged that vitamin D is one of the factors involved in the proliferation, differentiation, and regeneration of muscle cells [4]. As proof of this, alterations in vitamin D levels seem to be related to sarcopenia prevalence in several pathological conditions, including CD [5,6].

## 2. Sarcopenia and Crohn’s Disease

### 2.1. Definition of Sarcopenia

In the last decades, there has been a widespread interest in research about sarcopenia, whereby now it is formally recognized as a disease with an ICD-10-MC diagnosis code [7]. According to the latest definition provided by the European Working Group On Sarcopenia In Older People 2 (EWGSOP-2), sarcopenia has been defined as a progressive and generalized skeletal muscle disorder, associated with an increased probability of adverse outcomes, among which falls fractures, physical disability, and mortality [8].

This definition has evolved during the last years, with the addition of the muscle strength to the former definition based only on the muscle mass [9]. Indeed, according to the current knowledge, muscle strength seems to be a more reliable parameter to predict the adverse outcomes mentioned above [10,11]. Moreover, alterations in muscle strength seem to be related not only to changes in muscle quantity but also to deep alterations in muscle quality, caused by modifications in the architecture and composition of muscle cells [8]. According to this evidence, the latest guidelines raise muscle strength as the primary parameter to be evaluated for detecting sarcopenia [8].

### 2.2. The Assessment of Sarcopenia

Due to the well-established negative impact of sarcopenia on several pathological conditions, it should be mandatory for every clinician in charge of patients affected by chronic diseases, to evaluate and exclude the presence of sarcopenia, by means of validated case-finding tools.

Among these, the SARC-F questionnaire is the most used in daily clinical practice for patients aged ≥ 65 [12]. It is a self-reported questionnaire, with low sensitivity but high specificity, based on the patient’s self-evaluation of five motor abilities, i.e., walking, rising from a chair, climbing stairs, carrying weights, and avoiding falls [12]. An alternative recommended case-finding tool is the Ishii screening test, which relates age, handgrip strength, and calf circumference [13]. Nevertheless, including a skeletal muscle strength evaluation could be considered a more reliable tool to detect sarcopenia.

Should the screening tests return positive, evidence of low muscle quantity or low muscle quality by the use of tools available to this purpose would confirm a formal diagnosis of sarcopenia. In clinical practice, tool selection may depend on several variables related to both the patient and healthcare setting [8]. A brief overview of these tools is presented in Table 1.

### 2.3. Sarcopenia in Patients with CD

In the last decade, sarcopenia has emerged as a primary factor in the nutritional assessment of patients affected by chronic inflammatory diseases, including IBD [14,15]. In fact, there is evidence indicating that this syndrome impacts the course of the disease, the responsiveness to specific therapies, and the outcomes of surgery [14].

Sarcopenia turns out to be a widespread condition in patients with IBD, in particular CD [16]. In a recent systematic review, it is reported that up to 60% of patients with IBD present a depletion of the muscle mass when compared with healthy subjects [16].

It is reported that patients with CD, affected by sarcopenia, result to be overweight or obese (a condition named “sarcopenic obesity”), rather than undernourished, at the nutritional assessment tests [17]. This extreme variability emphasizes the need for malnutrition and sarcopenia screening in all CD patients.

**Figure 1:**
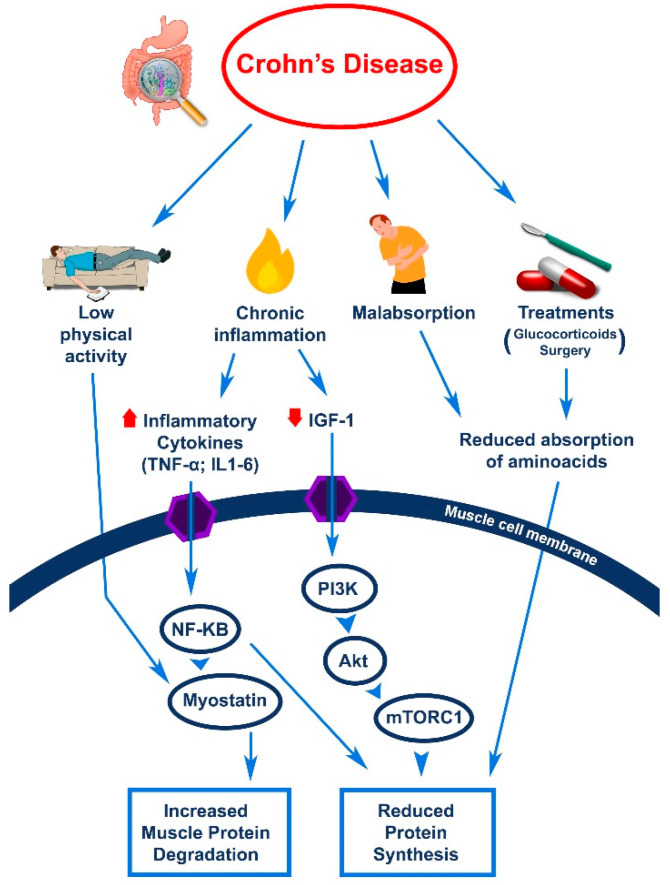
Different mechanisms are involved in the pathogenesis of sarcopenia in patients with CD. The combined effect of inflammatory cytokines (e.g., TNF-α, IL-6) in determining increased muscle protein degradation, through NF-KB and increased Myostatin activity, are here summarized. By reducing IGF-1 and its related intracellular signal pathway, along with the decreased amino acid absorption also due to therapeutic interventions, chronic inflammation determines a diminished protein synthesis. Abbreviations: TNF-α: tumor necrosis factor Alfa; IL-6: interleukin 6; PI3K: phosphatidylinositol-3-kinase; AKT: protein kinase B; mTORC1: mammalian target of rapamycin complex; IGF-1: Insulin-Like Growth Factor-1; NF-KB: Nuclear Factor Kappa B.

In addition to malabsorption and gastrointestinal surgery, other factors may contribute to the development of sarcopenia in patients with CD, such as eventual glucocorticoid treatment and hypogonadism, and a reduced physical activity [18,19,20]. It should be noted that the activation of inflammatory cytokines may contribute significantly to converting the muscle protein metabolism from synthesis to degradation, as shown in Figure 1 [18,21].

**Figure 2:**
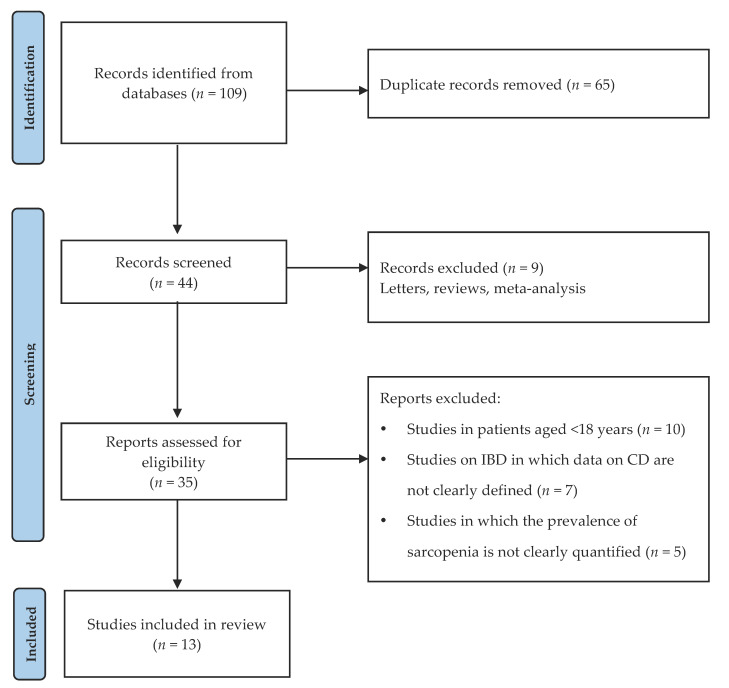
Flow diagram of the study selection process (adapted from PRISMA) [22].

To investigate the connection between sarcopenia and CD, we performed a systematic review, according to the Preferred Reporting Items for Systematic Reviews and Meta-Analyses (PRISMA) checklist [22], based on the following Medical Subject Heading (MeSH) keywords: “Crohn’s disease”, “inflammatory bowel disease”, “sarcopenia”. The search, performed on 10 March 2021, was made on the following on-line databases: Scopus (www.scopus.com) (1969–2020), MEDLINE (www.nlm.nih.gov) (1969–2020), and the US National Library of Medicine (www.PubMed.gov). Two authors (F.P. and R.D.T.) performed the screening of titles and abstracts. Full-length versions of selected articles were then assessed for inclusion criteria: studies in patients aged >18 years; diagnosis of CD performed according to the current international guidelines; papers published in English; studies on IBD in which data on CD were clearly defined; studies in which the prevalence of sarcopenia was clearly quantified. The study selection process is presented in Figure 2. The following data were collected: number of patients; variable, test, and tool used to assess sarcopenia in patients with CD; percentage of patients with sarcopenia (Table 2).

It has to be underlined that not all the above-cited studies were primarily focusing on the assessment of sarcopenia in patients with CD, which was instead part of a more extensive analysis. A common finding emerging from these studies, which were performed in different areas of the world, is the high prevalence of sarcopenia in patients with CD. The cohorts of these studies were heterogeneous and the percentage of patients with sarcopenia ranged from 19% to 61.4%, among different study populations. However, when considering only the data from the studies which used the gold standard tools (CT and MRI), the prevalence of sarcopenia increases significantly, ranging from 31% to 61.4%. Lastly, no significant difference in sample size, between studies using more expensive and time-consuming techniques (CT and MRI) and those that used more affordable tools (BIA and Dyn), is noteworthy.

Despite these described data, in daily clinical practice, the assessment of sarcopenia is still considered a marginal issue. Indeed, the most recent guidelines on CD do not mention the term “sarcopenia” [35].

## 3. Vitamin D Deficiency and Crohn’s Disease

### 3.1. Definition of Vitamin D Deficiency

Vitamin D is known to be produced in the skin from sunlight exposure or derived from foods that naturally contain vitamin D. According to the Endocrine Society guidelines, Vitamin D deficiency is defined as a serum 25-hydroxyvitamin D (25(OH)D) below 20 ng/mL (50 nmol/L), and vitamin D insufficiency as a serum 25(OH)D of 21–29 ng/mL (525–725 nmol/L) [36]. However, among scientific societies worldwide there is no general agreement on normal serum levels of 25(OH)D. Screening for vitamin D deficiency is indicated for all patients considered at risk, while population screening is not recommended [37]. In clinical practice, the most used way to evaluate vitamin D status is to determine serum concentrations of circulating 25(OH)D, measured with a reliable assay [36].

Vitamin D deficiency is acknowledged as a global health issue [38]. In addition to playing a crucial role in calcium and phosphorus homeostasis to preserve bone health, several studies have demonstrated a pleiotropic effect in different physiological processes. In particular, it has been recognized as a regulator of the innate immune system, of cardiovascular and renal functions, of cancer progression [37], and is also involved in different acute and chronic diseases [39].

### 3.2. Prevalence of Vitamin D Deficiency in Patients with CD

In the last decades, several studies have established the presence of vitamin D deficiency in patients with IBD, suggesting its potential role in the pathogenesis of these autoimmune diseases.

**Figure 3:**
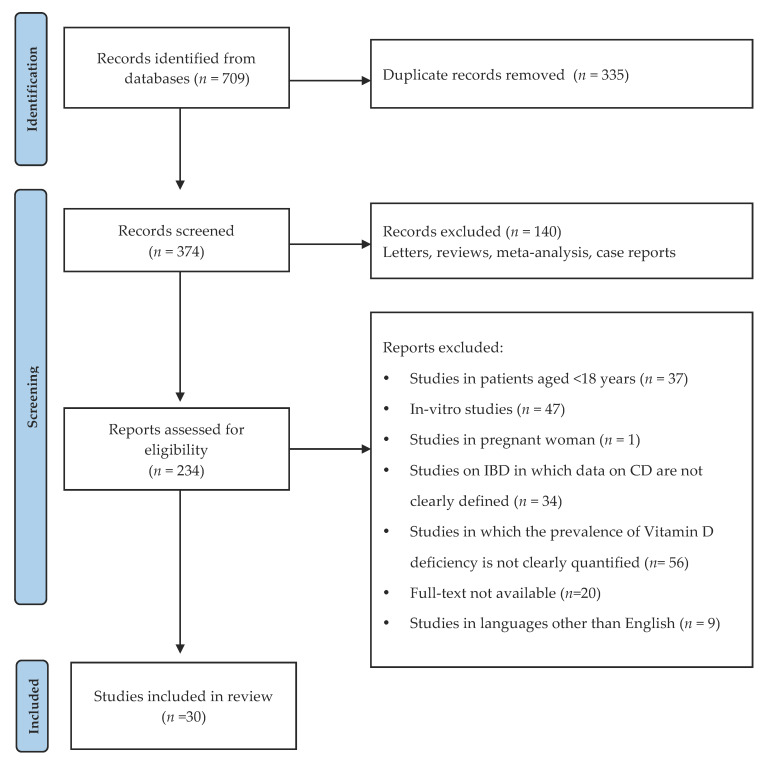
Flow diagram of the study selection process (adapted from PRISMA) [22].

As previously done for sarcopenia, we performed a systematic review to highlight the evidence that linked vitamin D deficiency to CD. The research was carried out according to the PRISMA checklist [22], on the online databases mentioned above and based on the following MeSH keywords: “Crohn’s disease”, “inflammatory bowel disease”, “vitamin D”, “cholecalciferol”, “25-hydroxyvitamin D”, “vitamin D deficiency”, “vitamin D status”. The analysis of the studies was performed by R.D.T. and F.P. The inclusion criteria were: studies in patients aged >18 years; diagnosis of CD performed according to the current international guidelines; diagnosis of vitamin D insufficiency or deficiency established for serum levels lower than 30 ng/mL; studies on IBD in which data on CD were clearly defined; studies in which the prevalence of vitamin D was clearly quantified; papers published in English. The following data were collected: number of patients; cut-off of 25(OH)D expressed in ng/mL adopted by the authors to define vitamin D deficiency; percentage of patients with vitamin D deficiency (Table 3). The study selection process is presented in Figure 3.

As shown in Table 3, all studies demonstrated vitamin D deficiency in patients with CD, ranging from 10.5% [49,59] to 100% [64], the majority showing high prevalence values.

It must be acknowledged that the majority of the studies in which vitamin D status was assessed were conducted on patients with IBD. To perform our review, we selected the studies in which the percentage of CD patients was clearly defined. 25(OH)D serum levels were expressed in ng/mL.

It should be specified that some authors provided different percentages based on the seasonal variability, with a lower prevalence of vitamin D deficiency occurring in summer than in winter [41,57,65,66], as expected.

Furthermore, recent data suggest that free 25(OH)D concentrations may be a better indicator than total 25(OH)D for the assessment of vitamin D status in patients with CD, due to the regulatory effects of glucocorticoid therapy and cytokines on vitamin D binding protein (VDBP) synthesis [70].

## 4. Vitamin D and Sarcopenia

### 4.1. Effects of Vitamin D on Skeletal Muscle Function

Over recent years, the potential role of vitamin D on muscle function and strength has been widely debated [4].

**Figure 4:**
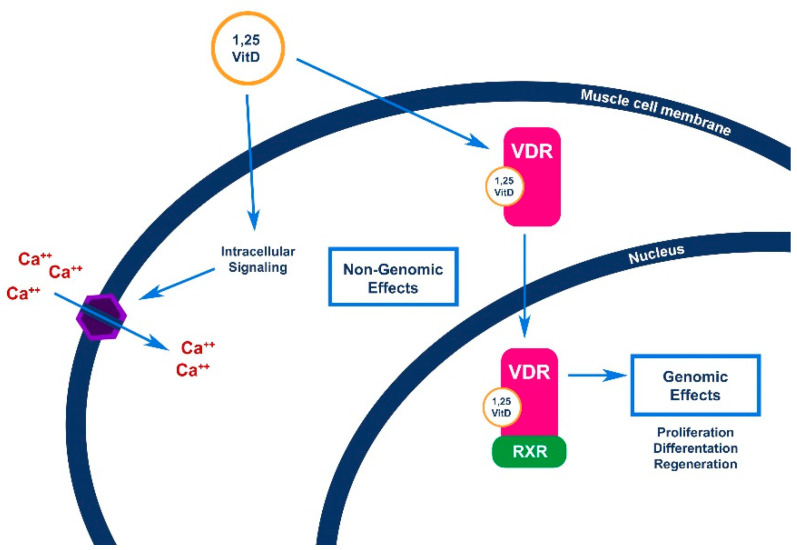
Brief overview of the genomic and non-genomic pathways of vitamin D at muscle cellular level. Vitamin D regulates gene expression in the nucleus by interacting with VDR, thus forming a heterodimeric complex of liganded VDR with RXR and upregulating or downregulating target genes transcription. The non-genomic effects of Vitamin D are mediated by the activation of several intracellular signal pathways through signal molecules, e.g., phospholipase C and phospholipase A2, and the production of second messengers, protein kinases, and the opening of Ca^2+^ and Cl^−^ channels. Abbreviations: VDR: Vitamin D Receptor; RXR: Retinoid-X-receptor; 1,25VitD: 1,25-hydroxyvitamin D.

At a cellular level, it is known that vitamin D acts through both genomic and non-genomic pathways, as summarized in Figure 4. At the nuclear level, vitamin D can regulate gene expression by interacting with Vitamin D Receptor (VDR), thus forming a heterodimeric complex of liganded VDR with Retinoid-X-receptor (RXR) and up-regulating or down-regulating target genes transcription. The non-genomic effects of Vitamin D are mediated by the activation of intracellular signal pathways through signal molecules, e.g., phospholipase C and phospholipase A2, and the production of second messengers, protein kinases, and the opening of Ca^2+^ and Cl^−^ channels as depicted in Figure 4.

Focusing on the biological mechanisms that regulate differentiation, proliferation, and regeneration of muscle cells, it has been demonstrated that vitamin D regulates several myogenic transcription factors involved in muscle cells proliferation, e.g., insulin-like growth factor 2 and follistatin [71], and in muscle cells differentiation, e.g., fetal myosin, the neural cell adhesion molecule, insulin-like growth factor 1, fibroblast growth factor and myogenic differentiation protein 1 [72,73].

Regarding muscle regeneration, it has been demonstrated that vitamin D promotes the initial increase of the cross-sectional area of skeletal muscle fibers, by arresting the cell cycle, and suppresses the expression of myostatin, a key factor implicated in muscular degeneration [74].

According to the current knowledge, vitamin D seems to mainly affect type IIA muscle cells, i.e., the “fast twitch oxidative” cells [71]. Indeed, by using muscle biopsy, in previous works it has been shown that vitamin D deficiency is associated with type IIA muscle cells atrophy and fibrosis [75] and, by contrast, the supplementation of vitamin D has been shown to increase the number and the diameter of type IIA muscle cells, thus increasing muscle strength [76].

Furthermore, it should be mentioned that elevated PTH may contribute to the pathogenesis of sarcopenia, given its direct effect on skeletal muscle protein metabolism and the recent demonstration that elevated PTH levels are associated with vitamin D deficiency in sarcopenia [77].

### 4.2. Vitamin D and Sarcopenia: Evidence from Other Patients

The relationship between vitamin D deficiency and sarcopenia has been extensively studied in other populations, and results from studies in these populations are interesting in regards to the potential role of vitamin D supplementation for the prevention and treatment of sarcopenia.

In particular, in the geriatric population, known to be at a high prevalence of vitamin D deficiency worldwide [78], a relationship between vitamin D and neuromuscular performance has been established [71,79].

Although the effects of the vitamin D on the intracellular nuclear and non-nuclear receptors to stimulate the growth and function of skeletal muscle cells has been well demonstrated in several studies, the clinical usefulness of oral vitamin D supplementation as a therapeutic mean to treat or prevent sarcopenia in older patients is still controversial [74].

This could interestingly be related to a decline in the VDR number with advancing age [80,81], thus making vitamin D supplementation probably more effective in younger patients.

It is worth noting that this specific issue has been the subject of a recent review within this journal and therefore will not be extensively discussed here [74].

## 5. The Missing Step: The Effect of Vitamin D Supplementation on Sarcopenia in Patients with CD

Our research aimed at finding relevant information in the scientific literature on the relationship between vitamin D supplementation and sarcopenia in patients with CD. However, in spite of the high prevalence of sarcopenia in patients with CD and the correlation between vitamin D metabolism and muscular performance, studies correlating these three clinical conditions are missing, although there is growing attention on this topic, especially among pediatric patients [82].

To the best of our knowledge, only Hradsky et al. performed a study on this issue, observing an improvement in muscle parameters after vitamin D supplementation in children with IBD, but without discrimination between CD and Ulcerative Colitis and using muscle strength as a variable to assess sarcopenia [83].

Given the role of vitamin D on muscle metabolism on a molecular basis, an improvement in sarcopenia in patients with CD could be expected with vitamin D supplementation. This open question requires further and appropriate studies.

## Figures and Tables

**Table 1:** Validated tests for the assessment of muscle strength and muscle quantity.

Variable	Parameter	Test	Tool	Advantages	Disadvantages
Skeletal Muscle Strength	GS	GST	Dynamometer	Simple and inexpensive.	Provides only an approximation for the strength of arm muscles. Not possible to perform in case of hands disability.
		CST	None	Simple and inexpensive.	Provides only an approximation for the strength of leg muscles. Not possible to perform in case of leg disability.
Skeletal Muscle Quantity	SMM	ASMI	BIA; DXA	Detailed information on the body composition Relatively low-cost method. Short time required.	Requires trained physicians Use of ionizing radiations (DXA)
	SMM	MCSA	CT; MRI	The gold-standard methods.	Requires highly trained personnel. Expensive tests. Time-consuming. Use of ionizing radiations (CT). Cut-off points for low muscle mass are not yet well defined.

Abbreviations: GS: grip strength; GST: grip strength test; CST: chair stand test; ASMI: appendicular skeletal muscle index; SMM: skeletal muscle mass; BIA: bioelectrical impedance analysis; DXA: Dual-energy X-ray absorptiometry; CT: computed tomography; MRI: magnetic resonance imaging; MCSA: muscle cross-sectional area.

**Table 2:** Prevalence of Sarcopenia in Crohn’s Disease (CD).

Authors	Year	*n*	Age (Years)	Variable	Test	Tool	Sarcopenia (%)
Boparai [23]	2021	44	34 ± 14.1 *	SMQ	MCSA	CT	43
Celentano et al. [24]	2020	31	46 (49–72) ^†^	SMQ	MCSA	MRI	38
Lee et al. [25]	2020	79	29 ± 11.3 *	SMQ	MCSA	CT	50
Grillot et al. [3]	2020	88	35 ± 12.4 *	SMQ	MCSA	CT	58
Thiberge et al. [26]	2018	149	41 ± 17.5 *	SMQ	MCSA	CT	33.6
Zhang T. et al. [27]	2017	105	−	SMQ	MCSA	CT	59
Csontos et al. [28]	2017	126	34 ± 11.5 *	SMQ	ASMI	BIA	29.4
Holt et al. [29]	2017	44	38 ± 14.2 *	SMQ	MCSA	CT MRI	41
Bamba et al. [30]	2017	43	29 (25–37) ^†^	SMQ	MCSA	CT	37
Cravo et al. [31]	2017	71	43	SMQ	MCSA	CT	31
Bryant et al. [32]	2015	95	31 (27–39) ^†^	SMQ SMS	ASMI GST	BIA Dyn	19 27
Zhang T. et al. [33]	2015	114	32 ± 11.5 *	SMQ	MCSA	CT	61.4
Schneider et al. [34]	2008	82	36 ± 13.9 *	SMQ	ASMI	DXA	60

Abbreviations: SMS: skeletal muscle strength; SMQ: skeletal muscle quantity; ASMI: appendicular skeletal muscle index; MCSA: muscle cross-sectional area; GST: grip strength test; Dyn: dynamometer; MRI: magnetic resonance imaging; CT: computed tomography; BIA: bioelectrical impedance analysis; DXA: Dual-energy X-ray absorptiometry; yr: year; − not found in the article; * the mean ± standard deviation; ^†^ the median range.

**Table 3:** Prevalence of Vitamin D deficiency in Crohn’s Disease (CD).

Authors	Year	n	Age (Years)	25(OH)D Cut-Off (ng/mL)	Vitamin D Deficiency (%)
Janssen et al. [40]	2019	256	43 (18–85) ^†^	<20 20–30	63% 24%
Burrelli Scotti et al. [41]	2019	33 78	-	<20	39.6% ^1^ 50% ^2^
Mentella et al. [42]	2019	101	37.9 ± 16.64 *	<20 <30	38.6% 25.7%
Frigstad et al. [43]	2018	227	40 (18–77) ^†^	<20	55%
Torella et al. [44]	2018	14	-	<30	78.6%
Lin et al. [45]	2018	346	-	<20	82.7%
Alrefai et al. [46]	2017	201	40 ± 15.2 *	<12 12–20	18% 26%
Venkata et al. [47]	2017	196	−	<30	58.7%
Pallav et al. [48]	2017	129	−	<20	40.3%
da Silva Kotze et al. [49]	2017	38	40 (16–73) ^†^	<20 20–30	10.5% 65.8%
Reich et al. [50]	2016	28	−	<30	53.6%
Rebouças et al. [51]	2016	75	41 ± 15.6 *	<30	62.7%
Xia et al. [52]	2016	124	27.6 ± 8.6 *	<20	67.8%
De Castro et al. [53]	2015	57	33 ± 9.8 *	<20 <30	33% 72%
Raftery et al. [54]	2015	119	45 ± 11.8 *	<20	36.1%
de Bruyn et al. [55]	2014	101	41 (30–50) ^†^	<20	54%
Dumitrescu et al. [56]	2014	14	36 ± 9 *	<20 <30	36% 43%
Hlavaty et al. [57]	2014	124 97	- -	<12	60% ^1^ 74% ^2^
Veit et al. [58]	2014	40	16.6 ± 2.2 *	<20	40%
Salacinski et al. [59]	2013	19	44 ± 10.3 *	<20 20–30	10.5% 37%
Fu et al. [60]	2012	40	40 ± 13.2 *	<20	42.5%
Suibhne et al. [61]	2012	81	36 ± 11 *	<20	63%
Atia et al. [62]	2011	43	61 ± 14.7 *	<20 <30	51.2% 83.7%
Jørgensen et al. [63]	2010	94	-	<20	30.9%
Kuwabara et al. [64]	2009	29	32 ± 6.7 *	<20	100%
Gilman et al. [65]	2006	58	38 ± 10.9 *	<20	19% ^1^ 50% ^2^
McCarthy et al. [66]	2005	44	37 ± 11.1 *	<20	18.2% ^1^ 50% ^2^
Tajika et al. [67]	2004	33	38 ± 7.5 *	≤10	27.3%
Siffledeen et al. [68]	2003	242	-	<10 <16	8% 22%
Jahnsen et al. [69]	2002	60	-	<12	27%

Abbreviations: yr: year; 25(OH)D: 25-hydroxyvitamin D; ^1^: percentage in summer; ^2^: percentage in winter; − not found in the article; * the mean ± standard deviation. ^†^ the median range.

## Data Availability

Statement excluded.
